# A randomized study comparing docetaxel/cyclophosphamide (TC), 5-fluorouracil/epirubicin/cyclophosphamide (FEC) followed by TC, and TC followed by FEC for patients with hormone receptor-positive HER2-negative primary breast cancer

**DOI:** 10.1007/s10549-020-05590-w

**Published:** 2020-03-13

**Authors:** Hiroshi Ishiguro, Norikazu Masuda, Nobuaki Sato, Kenji Higaki, Takashi Morimoto, Yasuhiro Yanagita, Makiko Mizutani, Shoichiro Ohtani, Koji Kaneko, Tomomi Fujisawa, Masato Takahashi, Takayuki Kadoya, Nobuki Matsunami, Yutaka Yamamoto, Shinji Ohno, Toshimi Takano, Satoshi Morita, Sachiko Tanaka-Mizuno, Masakazu Toi

**Affiliations:** 1 International University of Health and Welfare, 4-3 Kozunomori, Narita, 286-8686 Japan; 2 National Hospital Organization Osaka National Hospital, Osaka, Japan; 3 Niigata Cancer Center Hospital, Niigata, Japan; 4 Higaki Breastcare Clinic, Osaka, Japan; 5 Yao Municipal Hospital, Osaka, Japan; 6 Gunma Prefectural Cancer Center, Maebashi, Japan; 7 Hiroshima City Hiroshima Citizens Hospital, Hiroshima, Japan; 8 National Hospital Organization Hokkaido Cancer Center, Sapporo, Japan; 9 Hiroshima University Hospital, Hiroshima, Japan; 10 Shuto General Hospital, Yamaguchi, Japan; 11 Kumamoto University Hospital, Kumamoto, Japan; 12 Cancer Institute Hospital of JFCR, Tokyo, Japan; 13 Toranomon Hospital, Tokyo, Japan; 14 Kyoto University Hospital, Kyoto, Japan; 15 Shiga University of Medical Science, Otsu, Japan

**Keywords:** Breast cancer, Randomized clinical trial, Doxorubicin, Cyclophosphamide, Docetaxel, Neoadjuvant chemotherapy

## Abstract

**Purpose:**

Our primary objective was to determine the benefit/risk of anthracycline-free regimens by comparing docetaxel + cyclophosphamide (TC) alone, fluorouracil + epirubicin + cyclophosphamide (FEC) followed by TC, or TC followed by FEC as a primary treatment for patients with HR-positive, HER2-negative BC.

**Methods:**

We randomized patients with stage I–III HR-positive HER2-negative, operable BC to receive either six cycles of TC (TC6), three cycles of FEC followed by three cycles of TC (FEC-TC), or three cycles of TC followed by three cycles of FEC (TC-FEC). The primary endpoint was the pathological response. Secondary endpoints included clinical response, type of surgical procedure, recurrence, death, and adverse events (by NCI-Common Terminology Criteria for Adverse Events v.3.0). We conducted all statistical analyses using SAS Version 9.2.

**Results:**

We enrolled 195 patients and analyzed data from 193 as the intention-to-treat population. Pathological complete response rates were numerically higher in the TC6 group than in the other groups (*p* = 0.321). The breast conservation rate was significantly higher in the TC6 group (73%) than in the other groups (FEC-TC 51%, TC-FEC 45%, *p* = 0.007). Adverse events with grade > 3 were not common in the treatment groups (*p* = 0.569). The overall and distant disease-free survivals were similar among the groups with median follow-up of 5.80 years.

**Conclusions:**

Despite similar long-term efficacy and safety profile, the higher breast conservation rate in the TC6 group suggests that preoperative chemotherapy without an anthracycline may benefit patients with HR-positive HER2-negative BC.

**Trial registration:**

UMIN000003283 https://upload.umin.ac.jp/cgi-open-bin/ctr_e/ctr_view.cgi?recptno=R000003873.

**Electronic supplementary material:**

The online version of this article (10.1007/s10549-020-05590-w) contains supplementary material, which is available to authorized users.

## Background

Anthracycline–taxane sequential combination therapy is the standard perioperative chemotherapy regimen for breast cancer (BC) [1]. Strategies for perioperative chemotherapy are established on the basis of the subtypes of BC, namely, hormone receptor (HR)-positive [estrogen receptor (ER) and/or progesterone receptor-positive], human epidermal growth factor receptor 2 (HER2)-positive, and triple-negative. Although the prognosis of HR-positive BC is better than that of the other subtypes of BC [2–4], no standard chemotherapy has been established for HR-positive BC. The Japan Breast Cancer Research Group (JBCRG) has conducted a study to improve preoperative chemotherapy effectiveness in patients with stage I–III, HR-positive, and HER2-negative resectable primary BCs [5, 6]. The US Oncology Research Trial 9735 demonstrated that docetaxel + cyclophosphamide (TC) produced superior results over doxorubicin (also known as adriamycin) + cyclophosphamide (AC) in adjuvant settings [7]. Other studies suggested a benefit of anthracycline in higher-risk HR-positive disease but TC is appropriate option for lower-risk [8, 9].

Thus, taxane-based therapies combined with cyclophosphamide, rather than anthracyclines combined with cyclophosphamide, are attracting attention for BC treatment.

Our aim was to compare safety and efficacy among (1) six cycles of TC alone (TC6), (2) three cycles of TC followed by three cycles of FEC (TC-FEC), and (3) three cycles of FEC followed by three cycles of TC (FEC-TC) in patients assigned randomly to receive these therapies. The eligibility criteria included patients who had not undergone prior therapy and presented with resectable primary HR-positive, HER2-negative BC. Our primary objective was to identify the need for anthracycline in the preoperative treatment of luminal-type BC, as well as to confirm the importance of the order of the administration of chemotherapy agents. In addition, we assessed whether a direct correlation between efficacy and adverse events (AEs) (such as neutropenia) exists, and if such a correlation could serve as an index to predict treatment effectiveness.

## Methods

### Patients

We registered patients who fulfilled the eligibility criteria after they provided written informed consent to participate in the study. We filled a case registration form for each patient and faxed it to a case registration center in the Evidence-Based Medicine Research Center, Kyoto University Graduate School of Medicine, where it was checked and faxed back to each participating institution by the following business day. Patients began receiving treatment after the center confirmed the patient’s registration and regimen. This was a multicenter trial, and additional file lists the participating centers in Japan.

The inclusion criteria were as follows: patients aged 20–70 years with resectable primary invasive BC confirmed by needle or tissue biopsy (T1c-3N0-1M0); size of targe lesion ≤ 7 cm; HR-positive; HER2-negative; Eastern Cooperative Oncology Group performance status of 0 or 1; white blood cell count ≥ 4000/mm^3^ and ≤ 12,000/mm^3^, or neutrophil counts ≥ 2000/mm^3^; hemoglobin ≥ 9.0 g/dL; platelet count ≥ 100,000/mm^3^; aspartate aminotransferase (AST) and alanine transaminase (ALT) ≤ 2.5-fold the upper limits of normal (ULN); total/direct bilirubin at or below the ULN; serum creatinine ≤ 1.5-fold of the ULN; left ventricular ejection fraction ≥ 55%; lack of clinically significant abnormality on electrocardiogram; lack of interstitial pneumonia/pulmonary fibrosis by computed tomography imaging; and no prior therapy for BC.

We excluded patients with poorly controlled complications (malignant hypertension, myocardial infarction within the last 6 months, congestive cardiac failure) and those with pyrexia (suspected infection), bullous disease, pleural/pericardial effusions requiring treatment, severe edema, severe peripheral neuropathy, complications requiring steroid treatment, synchronous bilateral BC, history of invasive BC, and multiple cancers.

### Study design

**Fig. 1:**
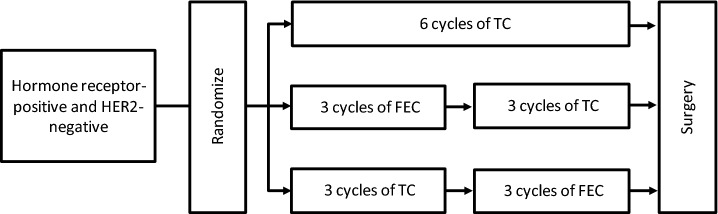
Study design. *TC* docetaxel + cyclophosphamide every 3 weeks, *FEC* 5-fluorouracil + epirubicin + cyclophosphamide every 3 weeks, *HER2* human epidermal growth factor receptor 2

Figure 1 shows the study design. We investigated three treatment regimens: (1) TC6: one cycle of TC therapy consisted of a 1-h intravenous (IV) infusion of docetaxel (75 mg/m^2^) followed by a 15–30-min IV infusion of cyclophosphamide (600 mg/m^2^); we administered 6 TC cycles to this group of patients. (2) FEC-TC: one cycle of FEC consisted of a 15–30-min IV infusion of 5-fluorouracil (500 mg/m^2^) followed by a bolus injection (within 5 min) of epirubicin (100 mg/m^2^) and then a 15–30-min IV infusion of cyclophosphamide (500 mg/m^2^); these patients received three cycles of FEC followed by three cycles of TC. (3) TC-FEC: we administered three cycles of TC followed by three cycles of FEC to this group of patients. We scheduled all chemotherapies to be given every three weeks.

### Criteria for therapy initiation and dose reduction

The criteria for starting each cycle included the following: neutrophil count ≥ 1,500/mm^3^; hemoglobin ≥ 8.0 g/dL; platelet count ≥ 75,000/mm^3^; AST and ALT ≤ 2.5-fold of the ULN; total/direct bilirubin ≤ 1.5-fold of the ULN; serum creatinine ≤ 1.5-fold of the ULN; non-hematological toxicity (excluding peripheral neuropathy, edema, and hair loss) ≤ grade 1; and peripheral neuropathy and edema ≤ grade 2. When the criteria were not fulfilled, treatment was postponed for up to 21 days until the symptoms resolved to grade ≤ 1.

During TC therapy, we allowed dose reductions by one level (docetaxel 60 mg/m^2^, cyclophosphamide 500 mg/m^2^), or two levels (docetaxel 45 mg/m^2^, cyclophosphamide 400 mg/m^2^). If further dose reductions were deemed necessary, we discontinued the investigational treatment. During FEC therapy, the doses could be reduced by one level (epirubicin 75 mg/m^2^), and we discontinued the investigational treatment if further dose reductions were deemed necessary.

### Premedication and supportive therapy

During TC therapy, when the patients were permitted to receive premedication one day before docetaxel administration, they received dexamethasone (4–8 mg) orally twice per day for 3 days. If premedication was not provided, dexamethasone (6.6–9.9 mg) was administered intravenously on the day of docetaxel administration. The use of antiemetics is by discretion of study investigators and the therapeutic use of only granulocyte colony-stimulating factor was allowed.

### Concomitant therapy

We prohibited concomitant use of therapy that might affect the assessments in this study (e.g. other antineoplastic agents, hormone therapy, biological response modifiers, radiotherapy, surgical therapy, and bisphosphonates). AEs were palliatively treated with the following medications: granulocyte colony-stimulating factor formulations for leukopenia or neutropenia; antihistamines, steroids, or preventive antibiotics for hypersensitivity, edema or infection; and omeprazole for gastrointestinal disorders.

### Surgery and postoperative therapy

The first operation was performed within 3–8 weeks of the last chemotherapeutic dose. Patients also received appropriate postoperative therapy, including chemotherapy and endocrine therapy.

### Endpoints

The primary endpoint was the pathological complete response (pCR) rate, defined as the proportion of patients who achieved comprehensive pCR (CpCR) for the primary lesion and pN0 or pN0(i+) for the axillary lymph node (yT0/isyN0). Secondary endpoints included clinical responses evaluated using diagnostic imaging techniques, such as CT or MRI, according to the Response Evaluation Criteria in Solid Tumors (RECIST) [10] and AEs evaluated using the Japanese JCOG (Japan Clinical Oncology Group) edition of the NCI-Common Terminology Criteria for Adverse Events v.3.0 (CTCAE v.3.0), and overall and disease-free survivals among patients who underwent operations.

### Histological assessment criteria

Histological effects were graded according to the “Criteria for Grading Histological Effect in Breast Cancer” and the “Criteria for Grading Histological Effect” of the “General Rules for Clinical and Pathological Recording of Breast Cancer, 16th Edition” by The Japanese Breast Cancer Society [11]. We categorized the pCR for primary lesions as follows: strict pCR (SpCR), pCR with in situ carcinoma (pCRinv), CpCR, near pCR, and quasi pCR (QpCR). For histological assessment of the axillary lymph nodes, we considered isolated tumor cells pN0(i+) of 0.2 mm or less were considered pN0.

### Target sample size

Overall, we aimed to enroll 195 individuals (65 per group). In other studies (JBCRG01 [5] and JBCRG03 [6]), CpCR in patients with HR-positive and HER2-negative BC was observed in 6% [95% confidence interval (CI) 3–12%] of those treated with FEC-T and in 13% (95% CI 6–23%) of those treated with T-FEC. Based on these results, we set the baseline probability of pCR in the present study at 9%. We considered a drug clinically useful when the pCR was at least 10% higher than the baseline probability. Therefore, assuming a difference in pCR rate among the TC6, FEC-TC and TC-FEC groups at 10% and a sample size of 195 subjects, with 65 subjects in each group, we calculated the probability of correctly selecting a treatment group with a high pCR rate to be 76.2%.

### Randomization

We randomized patients by stratification according to their ages (< 50 years vs. ≥ 50 years), metastasis to lymph node (N0 vs. N1), and treatment centers.

### Statistical analysis

For the primary endpoint pCR, we calculated the point estimation and two-sided 95% CIs. We made inter-group comparisons using the chi-square test. For the secondary endpoints, we used chi-square, Wilcoxon, and *t*-tests as appropriate. We analyzed toxicity by determining the incidence of AEs in each treatment group and then categorizing them according to grade. We made inter-group comparisons for grade 3–4 AEs and conducted all statistical analyses using the SAS version 9.2 software (SAS Institute, USA).

## Results

### Patients

**Fig. 2:**
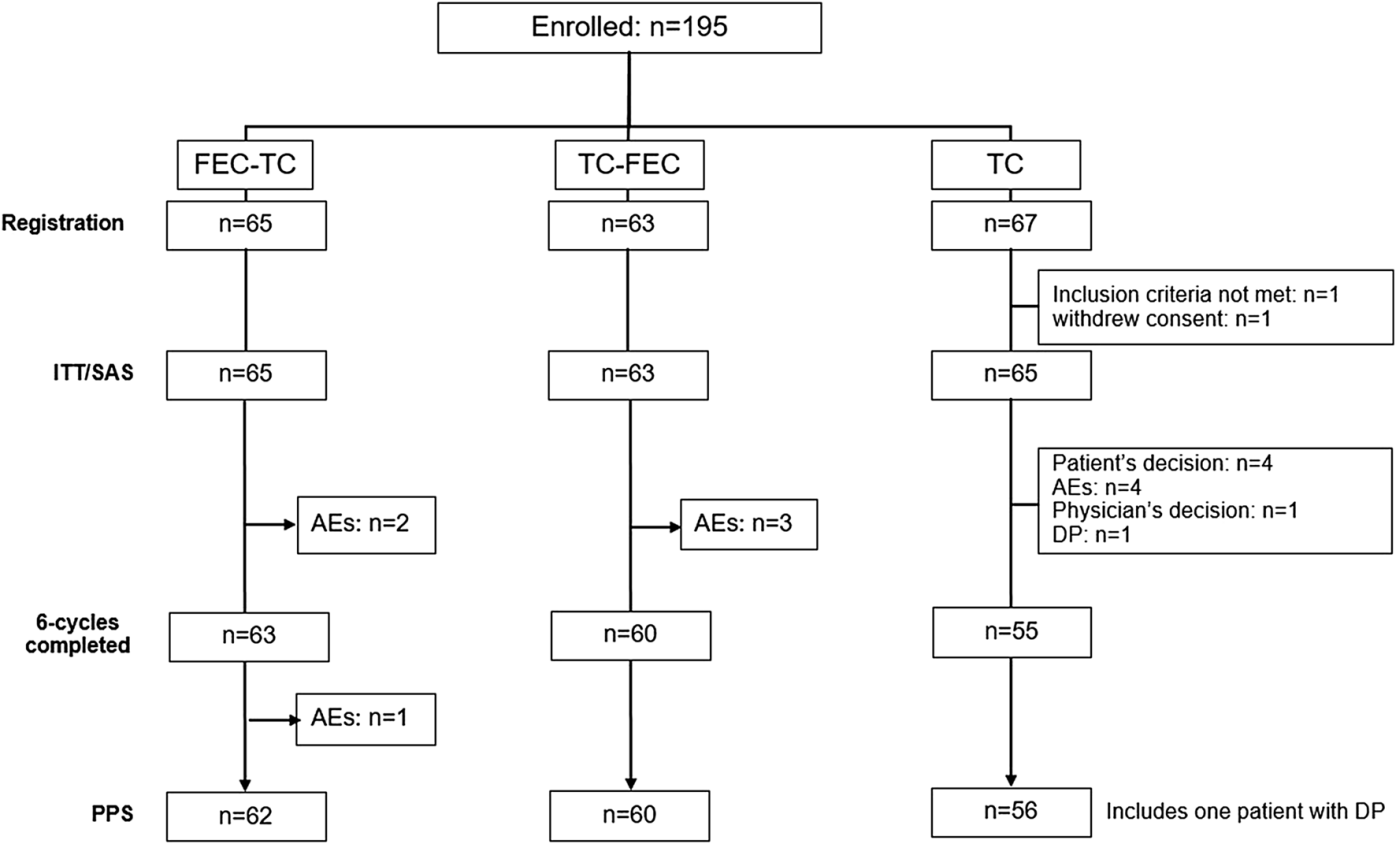
Study flowchart. *TC* docetaxel + cyclophosphamide, *FEC* 5-fluorouracil + epirubicin + cyclophosphamide, *AEs* adverse events, *PPS* per protocol set, *DP* disease progression, *ITT* intent-to-treat, *SAS* safety analysis set

We enrolled 195 patients between January 2010 and September 2011 by central registration and randomly assigned them to three groups. Figure 2 shows the study flowchart and Table 1 lists patients’ demographic characteristics. The median age was 49.5 years (range, 26–69 years), and 96% had BC stage II or higher. Among the 67 patients in the TC6 group, one withdrew consent before starting chemotherapy and one failed to meet the inclusion criteria; therefore, the TC6 group finally comprised 65 patients. The intent-to-treat (ITT) and safety populations comprised 193 patients (FEC-TC group, 65 patients; TC-FEC group, 63 patients; TC6 group, 65 patients), whose data were analyzed for treatment efficacy and safety according to the study protocol. The demographic characteristics of all the groups showed a similar distribution, with no major differences among the groups.

**Table 1:** Demographic characteristics of the patients

Number of patients (%)	Total	FEC-TC	TC-FEC	TC
	193	65	63	65
Age (years)				
Median (range)	48 (26–69)	50 (26–69)	48 (28–68)	50 (30–68)
cT				
cT1c	27 (14.0)	8 (12.3)	9 (14.3)	10 (15.4)
cT2	140 (72.5)	49 (75.4)	42 (66.7)	49 (75.4)
cT3	26 (13.5)	8 (12.3)	12 19.0)	6 (9.2)
Primary tumor (mm) by MRI or CT				
Median (range)	29 (9–82)	30 (10–70)	29 (10–70)	27 (9–82)
cN				
cN0	95 (49.2)	34 (52.3)	30 (47.6)	31 (47.7)
cN1	98 (50.8)	31 (47.7)	33 (52.4)	34 (52.3)
TNM				
I	8 (4.1)	2 (3.1)	3 (4.8)	3 (4.6)
IIA	97 (50.3)	35 (53.8)	28 (44.4)	34 (52.3)
IIB	71 (36.8)	23 (35.4)	25 (39.7)	23 (35.4)
IIIA	17 (8.8)	5 (7.7)	7 (11.1)	5 (7.7)
Menopause status				
Premenopausal	119 (61.7)	37 (56.9)	41 (65.1)	41 (63.1)
Postmenopausal	74 (38.3)	28 (43.1)	22 34.9)	24 (36.9)
Planed surgery				
Breast-conserving	114 (59.1)	37 (56.9)	36 (57.1)	41(63.1)
Mastectomy	79 (40.9)	28 (43.1)	2742.9)	24(36.9)
Histology				
IDC	182 (94.3)	58 (89.2)	60 (95.2)	64 (98.4)
ILC	9 (4.7)	6 (9.2)	2 (3.2)	1 (1.5)
Micropapillary	1 (0.5)	1 (1.5)	0	0
Medullary	1 (0.5)	0	1 (1.6)	0
IHC: ER				
≥ 80%	138 (71.5)	45 (69.2)	48 (76.2)	45 (69.2)
50%–79%	37 (19.2)	14 (21.5)	10 (15.9)	13 (20.0)
10–49%	13 (6.7)	4 (6.2)	4 (6.3)	5 (7.7)
1–9%	5 (2.6)	2 (3.1)	1 (1.6)	2 (3.1)
IHC: PgR				
≥ 80%	73 (37.8)	20 (30.8)	26 (41.3)	27 (41.5)
50–79%	37 (19.2)	18 (27.7)	9 (14.3)	10 (15.4)
10–49%	38 (19.7)	13 (20.0)	15 (23.8)	10 (15.4)
1–9%	23 (11.9)	9 (23.8)	4 (6.3)	10 (15.4)
0%	22 (11.4)	5 (7.7)	9 (14.3)	8 (12.3)
HER2				
IHC: 0	72 (37.3)	25 (38.5)	4 (6.3)	23 (35.4)
IHC: 1+	79 (40.9)	26 (40.0)	24 (38.1)	28 (43.1)
IHC 2+ or untested and FISH (−)	42 (21.8)	14 (21.5)	25 (39.7)	14 (21.5)

*IHC* immunohistochemistry, *FISH* fluorescence-based in situ hybridization, *ER* estrogen receptor, *PgR* progesterone receptor, *HER2* human epidermal growth factor receptor 2, *IDC* invasive ductal carcinoma, *ILC* invasive lobular carcinoma, *TC* docetaxel/cyclophosphamide, *FEC* 5-fluorouracil/epirubicin/cyclophosphamide

We discontinued the assigned chemotherapy treatment in three patients from the FEC-TC group due to AEs, in three patients from the TC-FEC group due to AEs, and in nine patients in the TC6 group due to AEs (*n* = 4), consent withdrawal (*n* = 4), and physician’s decision (*n* = 1).

### Efficacy

The pCR (CpCR + ypN0) rates were 9.2%, 8.1%, and 15.9% in the FEC-TC, TC-FEC, and TC6 groups, respectively (*p* = 0.321; Table 2). Table 3 shows the clinical tumor response data evaluated by CT and/or MRI. The ORRs were 72.8% in the FEC-TC group (CR 12.3%), 73.0% in the TC-FEC group (CR 4.8%), and 75.4% in the TC6 group (CR 15.4%).

**Table 2:** Results of pathological responses

	Total (*N* = 193)	FEC-TC (*N* = 65)	TC-FEC (*N* = 63)	TC (*N* = 65)	Comparative *p*-value across three groups
SpCR, *n* (%)	16 (8)	6 (9)	4 (7)	6 (10)	0.848
pCRinv, *n* (%)	11 (6)	3 (5)	4 (7)	4 (6)	0.861
Near pCR, *n* (%)	2 (1)	0 (0)	1 (2)	1 (2)	0.548
CpCR (SpCR + pCRinv), *n* (%)	27 (14)	9 (14)	8 (13)	10 (16)	0.901
QpCR (CpCR + near pCR, *n* (%)	29 (15)	9 (14)	9 (15)	11 (18)	0.876
CpCR + ypN0, *n* (%)	21 (11)	6 (9)	5 (8)	10 (16)	0.321
QpCR + ypN0, *n* (%)	23 (12)	6 (9)	6 (10)	11 (18)	0.280

*TC* docetaxel/cyclophosphamide, *FEC* 5-fluorouracil/epirubicin/cyclophosphamide, *pCR* pathological complete response, *SpCR* strict pCR, *pCRinv* in situ pCR, *CpCR* comprehensive pCR, *QpCR* quasi pCR

**Table 3:** Best overall response based on RECIST

	Total	FEC-TC group	TC-FEC group	TC group
*n* (%)	193 Patients	65 Patients	63 Patients	65 Patients
CR	21 (10.9)	8 (12.3)	3 (4.8)	10 (15.4)
PR	121 (62.7)	39 (60.0)	43 (68.3)	39 (60.0)
CR + PR	142 (73.6)	47 (72.3)	46 (73.0)	49 (75.4)
SD	40 (20.7)	14 (21.5)	15 (23.8)	11 (16.9)
PD	1 (0.5)	0	0	1 (1.5)
NE	10 (5.2)	4 (6.2)	2 (3.2)	4 (6.2)

*CR* complete response, *PR* partial response, *SD* stable disease, *PD* progressive disease, *NE* not evaluable, *TC* docetaxel/cyclophosphamide, *FEC* 5-fluorouracil/epirubicin/cyclophosphamide, *CT* computed tomography, *MRI* magnetic resonance imaging, *RECIST* Response Evaluation Criteria in Solid Tumors

Table 4 shows surgical procedures planned pre-chemotherapy performed in the ITT patient population according to the treatment arm. The rates of lumpectomy were 50.8%, 45.2%, and 73.0% in the FEC-TC, TC-FEC, and TC6 groups, respectively (*p* = 0.007). The proportions of patients scheduled to receive lumpectomy pre-chemotherapy who ultimately underwent mastectomy were 35.1%, 34.3%, and 10.0% in the FEC-TC, TC-FEC, and TC6 groups, respectively (*p* = 0.017). The proportions of patients scheduled to receive mastectomy pre-chemotherapy who ultimately underwent lumpectomy were 32.1%, 18.5%, and 43.5% in the FEC-TC, TC-FEC, and TC6 groups, respectively (*p* = 0.160).

**Table 4:** Types of planned and performed surgical operations and breast conservation rates

Pre-chemotherapy plan for surgical procedure	Surgery performed	Total (*N* = 193)	FEC-TC (*N* = 65)	TC-FEC (*N* = 63)	TC (*N* = 65)	Comparative *p*-value across three groups
Lumpectomy	Lumpectomy	83 (74.1%)	24 (64.9%)	23 (65.7%)	36 (90.0%)	
	Mastectomy	29 (25.9%)	13 (35.1%)	12 (34.3%)	4 (10.0%)	*p* = 0.017
	No surgery	2	0	1	1	
	Total	114	37	36	41	
Mastectomy	Lumpectomy	24 (30.8%)	9 (32.1%)	5 (18.5%)	10 (43.5%)	*p* = 0.160
	Mastectomy	54 (69.2%)	19 (67.9%)	22 (81.5%)	13 (56.5%)	
	No surgery	1	0	0	1	
	Total	79	28	27	24	
Lumpectomy		107 (56.3%)	33 (50.8%)	28 (45.2%)	46 (73.0%)	*p* = 0.007

Percentages were calculated excluding patients who did not undergo surgery

*TC* docetaxel/cyclophosphamide, *FEC* 5-fluorouracil/epirubicin/cyclophosphamide

**Fig. 3:**
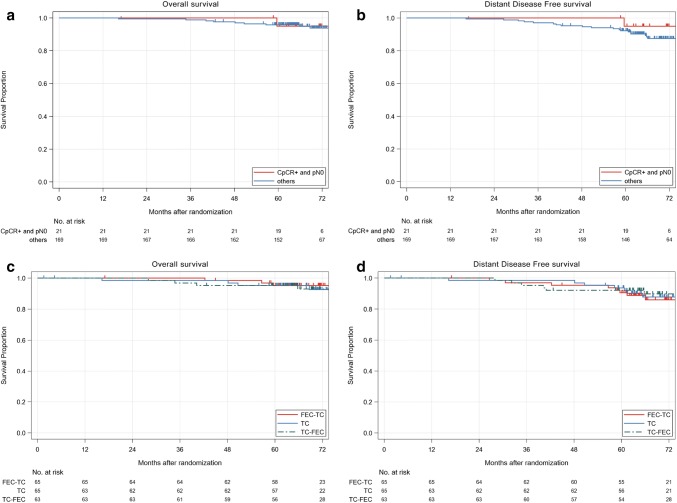
Efficacy results. Overall survival (**a**, **c**) and distant disease-free survival (**b**, **d**) differences between CpCR/pN0 and others (**a**, **b**) or among FEC-TC, TC-FEC or TC (**c**, **d**) in intent-to-treat population (*n* = 193). We excluded data from three patients who did not undergo surgical resection in **a** and **b**. *TC* docetaxel + cyclophosphamide, *FEC* 5-fluorouracil + epirubicin + cyclophosphamide

Figure 3 shows follow-up results. In the ITT population (*n* = 193), we found no differences between the FEC-TC, TC-FEC, and TC6 groups in terms of overall (*p* = 0.911) and distant disease-free survivals (*p* = 0.854), despite our relatively short median follow-up (5.80 years). Among the patients who completed surgery (*n* = 190), distant disease-free survivals were better in patients with pCR than in others, although the differences were not statistically significant (*p* = 0.356).

### Adverse events

Table 5 lists the AEs. The proportions of patients experiencing AEs of grade 3 or higher during the treatment period were 20.0%, 27.0%, and 20.3% in the FEC-TC, TC-FEC, and TC6 groups, respectively (*p* = 0.569). We observed grade 3 or higher febrile neutropenia in 18.5%, 22.2%, and 13.8% of patients in the FEC-TC, TC-FEC, and TC6 groups, respectively. Most patients were managed in an outpatient setting. AEs of grade 3 or higher other than febrile neutropenia (subjective and objective symptoms) included the following: allergic reactions or hypersensitivity; infection accompanied by grade 3 or 4 neutropenia; fatigue; skin eruptions or desquamation; inappetence; nausea; stomatitis; and edema of the trunk or genitals (Table 5). We encountered no adverse event-related deaths. Serious AEs occurred in four patients in the FEC-TC group (one patient developed right pulmonary arterial thrombosis, one calculous cholecystitis, and two with febrile neutropenia) and in four patients in the TC6 group (one with skin eruption/itching, one with pyelonephritis, one with gastritis, and one with exertional dyspnea/dry cough). We found no serious AEs in the TC-FEC group.

**Table 5:** G3 or 4 adverse events occurring in > 1 patient

	Total (*N* = 193)		FEC-TC (*N* = 65)		TC-FEC (*N* = 63)		TC (*N* = 65)	
	All grades	Grades 3/4	All grades	Grades 3/4	All grades	Grades 3/4	All grades	Grades 3/4
Subjective and objective symptoms								
Allergic reaction, hypersensitivity	16 (8.3)	2 (1.0)	3 (4.6)	0 (0.0)	10 (15.9)	1 (1.6)	3 (4.7)	1 (1.6)
Febrile neutropenia	35 (18.2)	35 (18.2)	12 (18.5)	12 (18.5)	14 (22.2)	14 (22.2)	9 (13.8)	9 (13.8)
Infection accompanied by grade 3/4 neutropenia	2 (1.0)	2 (1.0)	0	0	2 (3.2)	2 (3.2)	0	0
Fatigue (asthenia/inactivity/malaise)	106 (55.2)	1 (0.5)	38 (58.5)	0	34 (54.0)	1 (1.6)	34 (53.1)	0
Skin eruption, desquamation	96 (50.0)	2 (1.0)	20 (30.8)	0	36 (57.1)	0	40 (62.5)	2 (3.1)
Anorexia	86 (43.0)	1 (0.5)	31 (47.7)	1 (1.5)	28 (44.4)	0	27 (42.2)	0
Nausea	94 (49.0)	1 (0.5)	33 (50.8)	1 (1.5)	40 (63.5)	0	21 (32.8)	0
Stomatitis	84 (43.8)	2 (1.0)	30 (46.2)	0	29 (46.0)	1 (1.6)	25 (39.1)	1 (1.6)
Edema in the trunk or genitals	33 (17.5)	26 (13.8)	3 (4.7)	1 (1.6)	13 (21.0)	13 (21.0)	17 (26.9)	12 (19.4)
Stomatitis	84 (43.8)	2 (1.0)	30 (46.2)	0	29 (46.0)	1 (1.6)	25 (39.1)	1 (1.6)
Hematological tests								
White blood cell count	145 (75.5)	90 (46.9)	42 (64.6)	12 (18.5)	59 (93.7)	43 (68.3)	44 (68.8)	35 (54.7)
Neutrophil count	111 (58.4)	91 (47.9)	22 (33.8)	14 (21.5)	51 (82.3)	41 (66.1)	38 (60.3)	36 (57.1)
Hemoglobin	121 (63.0)	1 (0.5)	45 (69.2)	0	46 (73.0)	1 (1.6)	30 (46.9)	0 (0.0)
Platelet count	27 (14.1)	1 (0.5)	11 (16.9)	0 (0.0)	10 (15.9)	0 (0.0)	6 (9.4)	1 (1.6)
ALT	71 (37.0)	3 (1.6)	23 (35.4)	0 (0.0)	23 (36.5)	2 (3.2)	25 (39.1)	1 (1.6)

Data are reported as *n* (%)

*TC* docetaxel/cyclophosphamide, *FEC* 5-fluorouracil/epirubicin/cyclophosphamide, *ALT* alanine transaminase

## Discussion

In this study, we compared directly taxane-based therapies, with and without anthracyclines, for patients with primary HR-positive, HER2-negative BC, and we assessed the importance of the order of administration of chemotherapeutic agents. The incidences of severe AEs, grade 3 or higher, and the rates of study completion were similar among the groups. The most common adverse event was febrile neutropenia, confirming the findings of previous studies investigating FEC + docetaxel or docetaxel + FEC [5, 6]. Prophylactic use of granulocyte colony-stimulating factor was not available during the study period. The pCR rates in all three groups (TC6, TC-FEC, and FEC-TC) were similar. The breast conservation rate was significantly higher, and the proportions of patients scheduled to receive breast-conserving surgery, who ultimately received mastectomy, was significantly lower in the TC6 group than in the other groups. These findings suggest that six cycles of TC therapy may benefit patients who wish to undergo breast conservation treatment in the face of HR-positive, HER2-negative BC.

We are aware of the limitations in our study. First, the median follow-up period was relatively short for luminal-type BC, and we did not design the study to achieve statistical differences between the three treatment groups in terms of overall and distant disease-free survival. Long-term follow-up analyses are needed to investigate the anthracycline-free regimens as preoperative therapy for patients with early luminal-type BC. Moreover, a meta-analysis reported the lack of correlation between improved prognoses and higher pCR rates, especially among patients with HR-positive, HER2-negative BC [12]. Second, the availability of supportive care during the study period may have influenced the incidence of AEs. However, it was similar to that observed in clinical studies using a conventional TC regimen [7]. Third, our differences in the TC6 arm were not coupled with improved clinical response, which can be influenced by multiple factors like the tumor size, the location, distance from nipple, and/or patients’ preferences. Fourth, because the use of multigene assays, such as Oncotype Dx or MammaPrint, is not reimbursed in Japan, we were unable to exclude relatively low-risk patients who may not obtain survival advantage from our study, which might have interfered with the statistical power of this study. Some of these patients might still be sufficiently motivated to undergo lumpectomy surgery without survival advantage.

Previous trials in patients with BC showed that adding a taxane after administering an anthracycline-containing regimen improved patient pCR rates [13, 14]. The JBCRG 01 [5] and JBCRG 03 trials [6] showed the lowest pCR rates (14% and 27%, respectively) in the group containing patients with HR-positive, HER2-negative BC. Since using chemotherapy with anthracycline and taxane in these patients remains controversial, determining the most effective preoperative chemotherapy regimen for this subtype would be beneficial.

In a seven-year follow-up to US Oncology Research Trial 9735, comparing four cycles of standard-dose AC with the non-anthracycline regimen of TC alone in adjuvant settings, the TC group showed superior seven-year overall and seven-year disease-free survival rates than the AC group [7]. However, the same study demonstrated no effect of HR or HER2 status on treatment efficacy. On the other hand, the Joint Analysis of the ABC (Anthracyclines in Early Breast Cancer) Trials [USOR 06-090, NSABP B-46I/USOR 07132, NSABP B-49 (NRG Oncology)], including 2125 patients, comparing (TC) to anthracycline/taxane-based chemotherapy (TaxAC) indicated that the TaxAC regimens improved invasive disease-free survival (IDFS) in patients with high-risk human epidermal growth factor receptor 2-negative BC compared with the IDFS in those with TC6.

A review of adjuvant trials reported that administering a taxane, followed by an anthracycline (docetaxel followed by EC), provided greater drug dose intensity than an anthracycline followed by a taxane (FEC followed by docetaxel) [15]. Thus, taxane-first regimens might benefit BC patients. Also, JBCRG trials showed the incidence rate of grade 1/2 edema was lower (33%) after FEC when administered first followed by docetaxel (41%) than when administered in the inverse order [5, 6]. No serious AEs were reported in the TC-FEC group in this trial. These may suggest that docetaxel has a better safety profile when administered first.

## Conclusions

In summary, the efficacy and safety of six cycles of TC6 were equivalent to those of three cycles each of FEC + TC or of TC + FEC. The pCR rates in the three groups were similar, but the breast conservation rate was significantly higher in the TC6 group than in the others. Our results suggest that preoperative chemotherapy without an anthracycline may be introduced for patients with HR-positive, HER2-negative primary BC (especially when breast conservation is preferred).

## Electronic supplementary material

Below is the link to the electronic supplementary material.

Supplementary file1 (DOCX 12 kb)

## Data Availability

The data that support the findings of this study are available from the corresponding author upon reasonable request.

## Abbreviations

ABC: Anthracyclines in Early Breast Cancer
ALT: Alanine transaminase
AST: Aspartate aminotransferase
BC: Breast cancer
ER: Estrogen receptor
FEC: Fluorouracil/epirubicin/cyclophosphamide
HR: Hormone receptor
ITT: Intention-to-treat
JBCRG: Japan Breast Cancer Research Group
JCOG: Japan Clinical Oncology Group
RECIST: Response Evaluation Criteria in Solid Tumors
SAS: Safety analysis set
ULN: Upper limits of normal
