# Supplementary material for: A randomized study comparing docetaxel/cyclophosphamide (TC), 5-fluorouracil/epirubicin/cyclophosphamide (FEC) followed by TC, and TC followed by FEC for patients with hormone receptor-positive HER2-negative primary breast cancer
Source: Breast Cancer Res Treat. 2020 Mar 13;180(3):715–24. doi: 10.1007/s10549-020-05590-w (PMC7103001; doi:10.1007/s10549-020-05590-w)
Supplement: Supplementary file 1 — Supplementary file1 (DOCX 12 kb) [file 10549_2020_5590_MOESM1_ESM.docx]

**Additional file 1.** List of participating centers (Order based on enrolled patients’ numbers)

National Hospital Organization Osaka National Hospital

Niigata Cancer Center Hospital

Hiroshima City Hiroshima Citizens Hospital

Yao Municipal Hospital

Gunma Prefectural Cancer Center

National Hospital Organization Hokkaido Cancer Center

Hiroshima University Hospital

Osaka Rosai Hospital

Kumamoto University Hospital

Toranomon Hospital

National Hospital Organization Kyushu Cancer Center

Kyorin University Hospital

Yokohama Asahi Chuo General Hospital

Kyoto University Hospital

Osaka City University Hospital

Oikawa Hospital

National Nagasaki Medical Center

Nakagami Hospital

Komaki City Hospital

Hyogo College of Medicine
